# *Mycobacterium chelonae* infection of a cardiovascular bioprosthesis linked to a recent outbreak

**DOI:** 10.1007/s15010-025-02534-8

**Published:** 2025-04-07

**Authors:** Johanna Kessel, Axel Braner, Margo Diricks, Thomas Walther, Tomas Holubec, Rudolf Werner, Michael Hogardt, Thomas A. Wichelhaus, Stefan Niemann, Annette Moter, Inna Friesen, Nils Wetzstein

**Affiliations:** 1 Department of Internal Medicine, Infectious Diseases, Goethe University Frankfurt, University Hospital, Frankfurt am Main, Germany; 2 University Center of Infectious Diseases, UCI, Goethe University Frankfurt, University Hospital, Frankfurt am Main, Germany; 3 MVZ Aschaffenburg, Aschaffenburg, Germany; 4 Molecular and Experimental Mycobacteriology, Research Center Borstel, Borstel, Germany; 5 National and Supranational Reference Center for Mycobacteria, Research Center Borstel, Borstel, Germany; 6 German Center for Infection Research (DZIF), Partner Site Hamburg-Lübeck-Borstel-Riems, Borstel, Germany; 7 Department of Cardiovascular Surgery, University Cardiovascular Center Frankfurt am Main, Goethe University Frankfurt, University Hospital, Frankfurt am Main, Germany; 8 Department of Nuclear Medicine, Goethe University Frankfurt, University Hospital, Frankfurt am Main, Germany; 9 Institute of Medical Microbiology and Infection Control, Goethe University Frankfurt, University Hospital, Frankfurt am Main, Germany; 10 Biofilmcenter, Institute for Microbiology, Infectious Diseases, and Immunology, Charité - University Medicine Berlin and MoKi Analytics GmbH, Berlin, Germany; 11 Institute of Medical Microbiology and Virology, Leipzig University Hospital, Leipzig, Germany; 12 Moter Diagnostics, Berlin, Germany; 13 Mycobacterial Infection Research Unit (MIRU), Goethe University Frankfurt, University Hospital, Frankfurt am Main, Germany; 14 Department of Internal Medicine, Infectious Diseases, University Hospital Frankfurt, Theodor-Stern-Kai 7, 60590 Frankfurt, Germany

**Keywords:** NTM, Non-tuberculous mycobacteria, *Mycobacterium chelonae*, BioIntegral, Cardiac surgery, Mycobacterial infection, Bioprosthetic valve bearing conduit, Endocarditis

## Abstract

**Objectives:**

*Mycobacterium chelonae* is a rapid-growing non-tuberculous mycobacterium that has occasionally been described in connection with foreign material infections, e.g. after orthopaedic joint replacement or cosmetic surgery. In a recent outbreak, several cases of *M. chelonae* endocarditis associated with biological heart valve prostheses were reported.

**Case history:**

A 64-year-old female patient with a history of myalgia and recurrent joint swelling presented to our hospital. Initially suspected for rheumatoid arthritis, the patient underwent a series of orthopedic and rheumatologic treatments, including prednisolone and methotrexate. Subsequent history revealed a Ross operation in 2014 and a PET-CT was suspicious of a biological valved conduit infection leading to surgical replacement. Utilizing fluorescence in situ hybridization (FISH) diagnostic techniques, DAPI, Kinyoun and Ziehl-Neelsen staining, mycobacterial infection was confirmed in both the prosthesis and adjacent muscle tissue. Molecular methods identified a mycobacterium most closely related to the *M. chelonae/abscessus* complex indicating an association to a previously described outbreak of *M. chelonae* contaminated heart valves. Antimycobacterial therapy was initiated and the patient remains stable at the time of writing. To date, all mycobacterial cultures remained negative.

**Conclusions:**

Non-tuberculous mycobacteria (NTM) are rare and possibly underdiagnosed pathogens in infections of bioprosthetic flap bearing conduits. Mycobacterial foreign-body infections can manifest many years after implantation. As NTM can be difficult to detect, molecular identification methods are of particular importance. Here, modern imaging, molecular and microscopic techniques might be of special use in diagnosing prolonged prosthetic graft infections.

## Case description

**Fig. 1:**
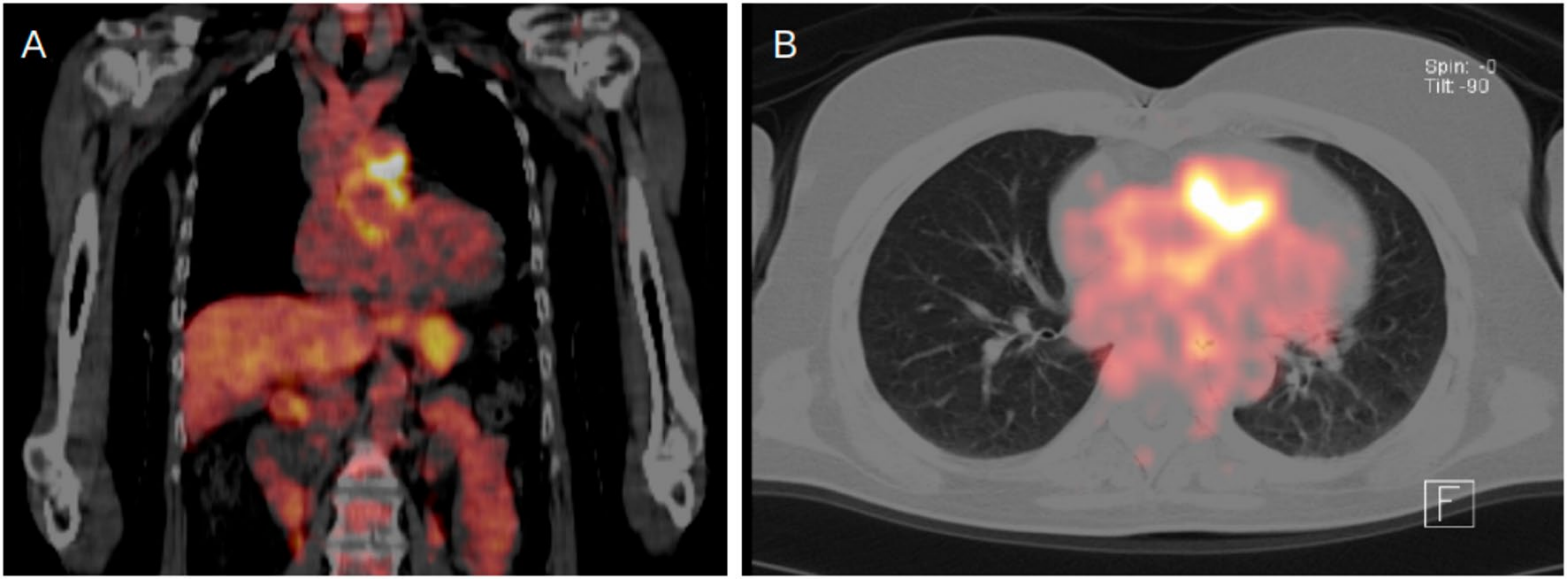
FDG-PET-CT scan indicating prosthetic graft infection in pulmonary position (frontal plane -**A**; horizontal plane -**B**), FDG-PET-CT - 18 F-fluorodeoxyglucose positron emission tomography-computed tomography

A 64-year-old female patient with a two-year history of myalgia and recurrent swelling of various joints presented to our rheumatology department in May 2023. A previous rheumatological work-up (including a FDG-PET-CT showing signs of large vessel vasculitis) led to the suspicion of late onset rheumatoid arthritis with polymyalgia rheumatica aspect. After exclusion of a latent tuberculosis infection, medical treatment with prednisolone and methotrexate was initiated before admission but did not lead to substantial symptomatic improvement. Her medical history included a Ross operation in 2014, in which the pulmonary valve and root were replaced using a biological valved conduit (BioIntegral Surgical Inc, Mississauga, ON, Canada, formerly Shellhigh). On suspicion of giant cell arteritis, another FDG-PET-CT scan was performed, which in contrast to the examination a year earlier, provided no evidence of arteritis, but showed circular, wall-like tracer enhancement in the area of the xenograft (Fig. 1).

**Fig. 2:**
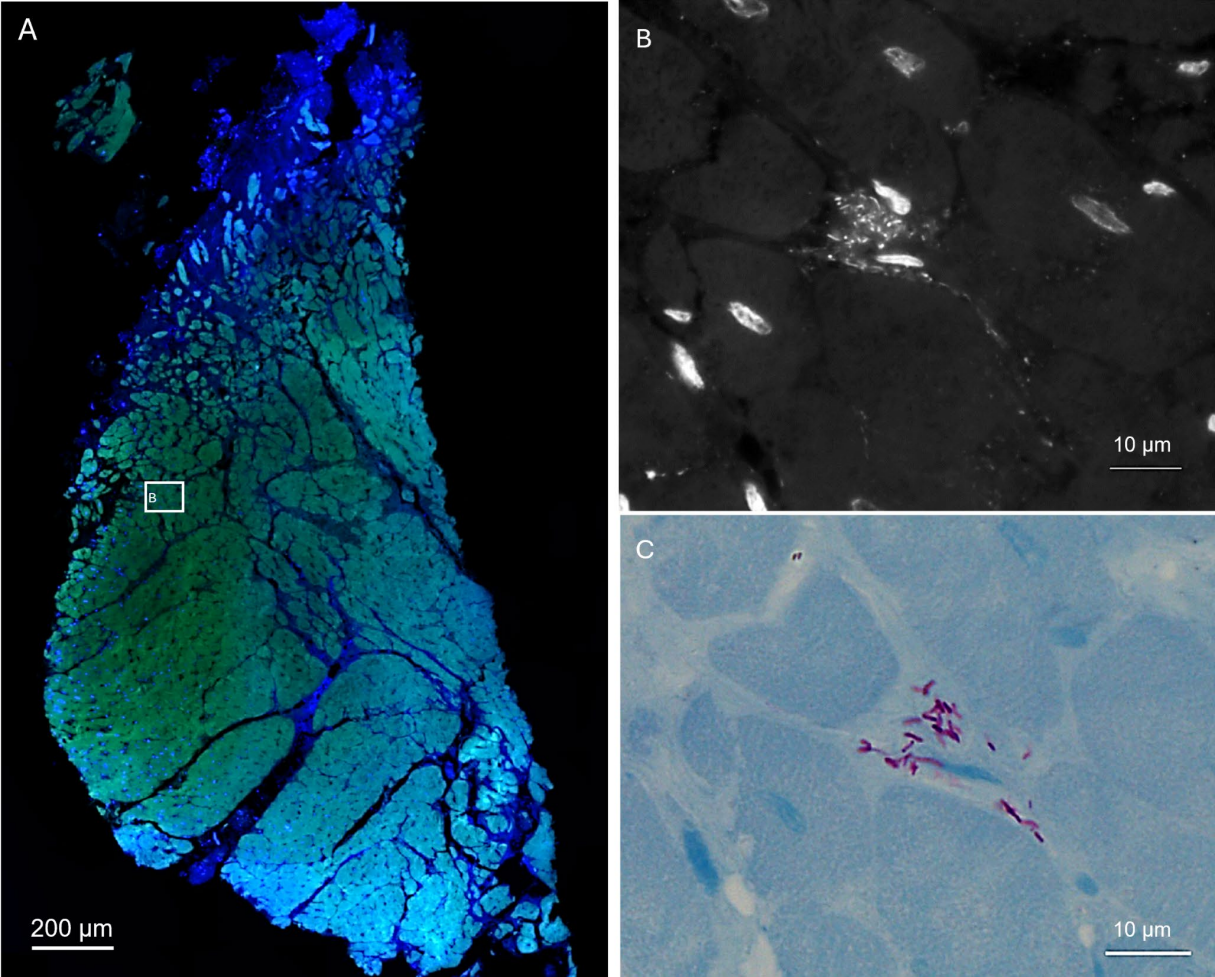
Fluorescence in situ hybridization on tissue sections of removed cardiac tissue shows bacterial colonization of muscle tissue (A), overview shows the filter sets for the nucleic acid stain DAPI (blue) staining host cell nuclei and the autofluorescent tissue background in green. The inset (B) marks the region that is further inspected at higher magnification. At higher magnification numerous rods are visible in spaces between and around muscle cells, as shown by nucleic acid stain DAPI in black and white. No FISH signals were detected with a pan-bacterial or NTM-specific FISH probe, indicating a low ribosome content of the bacteria (data not shown), (C) shows the identical location stained with Kinyoun reveals acid-fast bacilli. DAPI – 4′,6-diamidino-2-phenylindole; FISH – Fluorescence in situ hybdridization; NTM – non-tuberculous mycobacteria

Due to suspected prosthesis infection, the patient was scheduled for reoperation. The foreign material was completely removed and the pulmonary root was re-replaced by a pulmonary homograft in January 2024. Conventional culture from the removed material remained negative. However, histopathological examination revealed necrosis with focal inflammation in the removed conduit. Ziehl-Neelsen and Auramin staining provided microscopic evidence of acid-fast bacilli in the patient’s muscle tissue. Fluorescence in situ hybridization (FISH) diagnostic, as well as 4′,6-diamidino-2-phenylindole (DAPI) and Kinyoun staining of cardiac tissues were performed at the Biofilm Laboratory of the Charité, Berlin, Germany [1]. Although, peptide nucleic acid probes-(PNA)-FISH remained negative (Fig. 2A), DAPI staining could demonstrate numerous DNA-positive rods (Fig. 2B) [2]. These were located between cardiac muscle cells and destructed areas within the tissue. Further, Kinyoun staining showed acid-fast bacilli in the same locations (Fig. 2C). Thus, the presence of mycobacteria in the prosthesis, as well as in adjacent muscle (possibly linked to slow growth into the tissue) was confirmed.

Further molecular methods for the identification of mycobacteria involved the Genotype CMdirect V1.0 line probe assay (Hain Lifescience, Germany) according to the manufacturer’s instructions as well as amplification and sequencing of the intergenic transcribed spacer (ITS) region of the 16–23 S rRNA gene with primers targeting the 3´ end of the 16 S rRNA gene (ITS1, 5-GATTGGGACGAAGTCGTAAC) or the 5´ end of the 23 S rRNA gene (ITS2, 5-AGCCTCCCACGTCCTTCATC) as described by Richter et al. [3] (conducted at Frankfurt University Hospital and the National Reference Center for Mycobacteria, Borstel, Germany). The bacterium was identified to be closely related to the *Mycobacterium abscessus/chelonae* complex by Genotype CMdirect. The Genotype NTM-DR (Hain Lifescience, Germany) revealed a wildtype of the *rrl* gene and therefore no evidence for constitutional macrolide resistance. Amplification of the *rrs* gene for the genotypical testing of aminoglycoside susceptibility was unsuccessful. Whole genome sequencing was not possible due to low DNA concentrations.

Mycobacterial cultures were performed using BD BACTEC™ MGIT™, Löwenstein-Jensen, and Middlebrook agar plates incubated at 30°, 37° and 42° temperature. To this date, all cultures remained sterile. Based on the origin of the prosthesis, we considered *M. chelonae* the most likely causal pathogen.

To prevent infection of the novel prosthesis, antimycobacterial treatment with azithromycin 500 mg OD p.o., clofazimine 100 mg OD p.o., linezolid 600 mg OD p.o., imipenem 1000 mg TID i.v, and amikacin 750 mg i.v. OD was started two days after surgery in January 2024. Amikacin was later exchanged for tobramycin. This initial therapy was subsequently tapered to an all-oral regimen containing azithromycin, clofazimine, and linezolid. After four weeks the patient was discharged. Therapy was tolerated reasonably well, but significant QTc prolongation occurred as a relevant side effect of azithromycin. In the ninth week of therapy, the patient was readmitted to the hospital due to dyspnea and weakness with cardiac insufficiency (NYHA class III) and right-sided pleural effusions. Echocardiography demonstrated good prosthesis function. A cardiac MRT confirmed a severely impaired left ventricular pump function and provided evidence of intramyocardial scarring of the lateral ventricular wall. A cardiac biopsy was performed, but the histological examination did not indicate the potential presence of mycobacterial infection or signs of inflammation. However, hsp65-PCR from formalin embedded material was positive for non-tuberculous mycobacteria (closest relative: *M. phocaicum*). Nevertheless, in the absence of inflammatory signs on histology, dilative cardiomyopathy was considered the most probable diagnosis.

Following the initiation of heart failure therapy, the patient’s symptoms improved rapidly, allowing for continuation of antimycobacterial therapy on an outpatient basis. At the time of writing, the condition is stable after 12 months of therapy (currently with azithromycin and clofazimine).

## Discussion

*M. chelonae* is a rapid-growing non-tuberculous mycobacterium that has occasionally been described in connection with foreign material infections, e.g. after orthopaedic joint replacement or breast implants [4, 5]. The first case of *M. chelonae* endocarditis in connection with biological valved conduit from the manufacturer BioIntegral Surgical Inc was described in 2020 [6]. Since then, several cases of bioprosthestic endocarditis in products from this manufacturer have been reported, in which evidence of colonization with *M. chelonae* was found after explantation of these valved bioprostheses (6, 7). A recent investigation could detect *M. chelonae* only in BioIntegral products and not in 228 bioprostheses supplied by other manufacturers [8]. To date, all cases have been detected by microscopy and/or molecular biology alone, and it has not been possible to culture the pathogen.

Consequently, in July 2022, the manufacturer terminated all certificates required for market access in the EU, meaning that no new products may be placed on the market in the EU. Prostheses on the market should not be implanted [9]. Unfortunately, it can be assumed that some contaminated prostheses could still lead to clinically apparent infections in situ, and that as in our case, these will be misdiagnosed, diagnosed late, or possibly remain undiagnosed. Contrary to most published cases [6, 10], our patient developed symptoms only seven years after surgery and was diagnosed after even ten years. 

To conclude, non-tuberculous mycobacteria (NTM) are rare and possibly underdiagnosed pathogens in infections of bioprosthetic valved conduits. Mycobacterial foreign-body infections can manifest many years after implantation. As a result, there is often a delay of months to years between the onset of clinical symptoms and diagnosis [11]. Symptoms of disseminated disease might be myalgia and joint pain and can be mistaken for rheumatic disease, as was the case in our patient. As NTM are difficult to detect and special culture media are required for their cultivation, molecular detection methods are of particular importance for their identification and specification. Modern imaging, molecular and microscopic techniques might be of special use in diagnosing prolonged prosthetic graft infections.

## Data Availability

No datasets were generated or analysed during the current study.

## Abbreviations

DAPI: 4',6-diamidino-2-phenylindole
FISH: Fluorescence *in situ* hybdridization
FISHseq: Fluorescence *in situ* hybridization combined with 16SrRNA-gene PCR and sequencing of subsequent tissue sections
NTM: Non-tuberculous mycobacteria
NYHA: New York Heart Association
OD: Once daily
FDG-PET-CT: 18 F-fluorodeoxyglucose positron emission tomography-computed tomography
PNA: Peptide nucleic acid probes
PCR: Polymerase chain reaction
TID: Ter in die/three times a day
rRNA: ribosomal ribonucleic acid
